# Synergistic effects of *Moringa oleifera* and *Thymus vulgaris* methanolic extracts on growth performance, Gut acid-base balance, Humoral immunity, and nutrient digestibility in broiler chickens

**DOI:** 10.1016/j.psj.2026.106423

**Published:** 2026-01-11

**Authors:** Majid Ali, Hanan Alkhalaifah, Naila Chand, Shabana Naz, Rifat Ullah Khan, Ala Abudabos, Kasim Sakran Abass, Sohail Ahmad, Ibrahim A. Alhidary

**Affiliations:** a Department of Poultry Science, Faculty of Animal Husbandry and Veterinary Sciences, The University of Agriculture, Peshawar, Pakistan; b Environment and Life Sciences Research Center, Kuwait Institute for Scientific Research, Safat, Kuwait; c Department of Zoology, Government College University, Faisalabad, Pakistan; d Physiology Lab, College of Veterinary Sciences, Faculty of Animal Husbandry and Veterinary Sciences, The University of Agriculture, Peshawar, Pakistan; e Department of Food and Animal Sciences, College of Agriculture, Tennessee State University, Nashville, TN 37209 USA; f Department of Physiology, Biochemistry, and Pharmacology; College of Veterinary Medicine; University of Kirkuk, Kirkuk 36001, Iraq; g Islamic University of Afghanistan, Kabul, Afghanistan; h Department of Animal Production, College of Food and Agriculture Science, King Saud University, Riyadh, Saudi Arabia

**Keywords:** *Moringa oleifera*, *Thymus vulgaris*, Broilers, Growth performance, Immune response

## Abstract

This study evaluated the effects of **methanolic extracts of *Moringa oleifera* (MO) and *Thymus vulgaris* (TV), administered alone or in combination via drinking water**, on growth performance, carcass traits, gut health, immune response, lymphoid organ development, liver and kidney function, and nutrient digestibility in broiler chickens. A total of **540 one-day-old Ross 308 broiler chicks were randomly allocated to six dietary treatments for a 42-day experimental period**, including **a basal diet control, a basal diet supplemented with a standard antibiotic growth promoter, MO extract alone, TV extract alone, and two combined MO–TV extract treatments. The combined treatments consisted of graded inclusion levels, with the most effective group receiving M50T50 (100mg/L MO + 150mg/L TV)**.

The results demonstrated that **combined MO and TV supplementation**, particularly **M50T50**, significantly improved feed intake, body weight gain, and feed conversion ratio compared with the **control and single-extract groups** (*p* = 0.01). Dressing percentage and breast and thigh yields were highest in the **combined extract groups** (*p* = 0.01). **Intestinal pH was significantly reduced**, indicating an improved gut environment (*p* < 0.05), while **antibody titers against infectious bronchitis (IB), Newcastle disease (ND), and infectious bursal disease (IBD) viruses were markedly elevated** (*p* < 0.01). **Lymphoid organ development, particularly bursal weight, was enhanced**, whereas liver and kidney function markers remained within normal physiological ranges. **Crude protein digestibility was also significantly increased** (*p* = 0.01).

These findings **demonstrate that combined MO and TV methanolic extracts exert synergistic effects** on growth performance, immunity, gut health, and protein utilization in broilers without adverse effects, **highlighting their potential as a natural and effective phytogenic alternative to antibiotic growth promoters in poultry production**.

## Introduction

The rapid expansion of the poultry industry has increased the demand for safe, efficient, and sustainable feed additives that can enhance productivity and immune competence while reducing dependency on synthetic growth promoters (Rahman et al., 2017; Chand et al., 2018; Rasool et al., 2024; Sultan et al., 2025). Among natural alternatives, phytogenic feed additives derived from medicinal plants have gained significant attention for their antimicrobial, antioxidant, and immunomodulatory properties (Hafeez et al., 2020; Khan et al., 2021; Ullah et al., 2022). *Moringa oleifera,* commonly known as the drumstick or miracle tree, is rich in vitamins, minerals, essential amino acids, and bioactive compounds such as flavonoids, tannins, and saponins (Mahfuz and Piao, 2019; Bashir et al., 2024; Salam et al., 2025). Several studies have reported that supplementation of *M. oleifera* leaf extract or powder improves broiler growth performance, nutrient digestibility, antioxidant status, and intestinal microbial balance (Jiang et al., 2023; Akib et al., 2024). In addition, *M. oleifera* possesses immunostimulatory effects, enhancing antibody titers and lymphoid organ development in chickens (Tolba et al., 2022).

Similarly, *Thymus vulgaris* (thyme) essential oil is well-documented for its high phenolic content, primarily thymol and carvacrol, which exhibit potent antimicrobial and antioxidant activities (Zaazaa et al., 2022). Dietary inclusion of thyme or its essential oil has been shown to improve feed efficiency, carcass quality, and oxidative stability of broiler meat while reducing harmful gut bacteria (Gümüş and Gelen, 2023). Thyme also modulates the immune response by influencing cytokine production and enhancing humoral immunity (Popović and Puvača, 2019).

Although the beneficial effects of *M. oleifera* and *T. vulgaris* have been extensively studied individually, **information regarding their combined application, particularly in methanolic extract form, remains limited**. Importantly, the potential **synergistic interactions between their polyphenolic, antimicrobial, and immunomodulatory compounds have not been systematically evaluated** in broiler chickens. Furthermore, few studies have simultaneously examined growth performance, gut pH modulation, nutrient digestibility, and humoral immunity within a single experimental framework.

Therefore, the present study was designed to test the hypothesis that combined methanolic extracts of *M. oleifera* and *T. vulgaris* exert synergistic effects on broiler performance by enhancing digestive efficiency, stabilizing gut pH, and strengthening humoral immune responses beyond what is achievable with individual plant supplementation. This integrated approach allows for a more comprehensive understanding of phytogenic synergy and provides novel insights into the strategic use of combined plant extracts as functional feed additives in poultry nutrition.

## Materials and methods

### Preparation of *T. vulgaris* and *M. oleifera* methanolic extracts

Fresh leaves of *T. vulgaris* and *M. oleifera* were collected from Khyber Pakhtunkhwa, Pakistan, and authenticated by the Department of Horticulture, The University of Agriculture, Peshawar (voucher Nos. MO-KP-2022-01 and TV-KP-2022-02). Leaves were shade-dried for five days, ground, and sieved (0.15 mm). Each powder (400 g) was Soxhlet-extracted with 2 L of methanol (99.8%), filtered, and concentrated using a rotary evaporator (35°C, 150 rpm). Phytochemical screening for alkaloids, flavonoids, phenolics, tannins, and saponins followed Harborne (1998). Total phenolic and flavonoid contents were quantified by Folin–Ciocalteu and aluminum chloride assays. Extracts were sterilized (0.22 µm filters) and stored at 4°C until use.

### Phytochemical characterization of *M. oleifera* and *T. vulgaris* methanolic extracts

Dried leaves of *M. oleifera* and aerial parts of *T. vulgaris* were finely ground and subjected to methanolic extraction. Briefly, 50 g of each powdered plant material was extracted with 500 mL of 80% methanol using a Soxhlet apparatus for 6 h. The extracts were filtered and concentrated under reduced pressure using a rotary evaporator at 40°C, then stored at −20°C until analysis. Total phenolic content (TPC) was determined using the Folin–Ciocalteu method and expressed as mg gallic acid equivalents (GAE)/g extract. Total flavonoid content (TFC) was measured using the aluminum chloride colorimetric method and expressed as mg quercetin equivalents (QE)/g extract. Antioxidant activity was evaluated using the DPPH radical scavenging assay. Identification and quantification of major bioactive compounds in *T. vulgaris* extract (thymol, carvacrol, and β-caryophyllene) were performed using gas chromatography–mass spectrometry (GC–MS), while key phenolic constituents of *M. oleifera* extract (including quercetin, kaempferol, and chlorogenic acid) were analyzed by high-performance liquid chromatography (HPLC) following standard procedures described previously in the literature (Table 1).

**Table 1:** Phytochemical composition and antioxidant activity of plant extracts.

Parameter	*Moringa oleifera* extract	*Thymus vulgaris* extract
Total phenolics (mg GAE/g extract)	82.4 ± 3.1	96.7 ± 2.8
Total flavonoids (mg QE/g extract)	41.6 ± 1.9	38.2 ± 1.5
DPPH inhibition (%)	68.5 ± 2.4	74.9 ± 2.1
Thymol (% of total volatiles)	ND	41.3
Carvacrol (% of total volatiles)	ND	18.6
β-caryophyllene (%)	ND	6.8
Quercetin (mg/g extract)	9.4	ND
Chlorogenic acid (mg/g extract)	6.7	ND

Values are expressed as mean ± SEM. ND: not detected.

### Experimental design and treatment protocol

A total of 540 day-old Ross 308 broiler chicks of uniform body weight and health status were procured from a local commercial hatchery and used in this experiment to evaluate the effects of *M. oleifera* and *T. vulgaris* methanolic extracts, either singly or in combination, on broiler performance. Upon arrival, chicks were individually weighed, wing-banded for identification, and randomly distributed into six dietary treatment groups under a completely randomized design (CRD). Each treatment group consisted of six replicates, with 15 chicks per replicate (*n* = 90 birds per treatment).

All birds were housed in floor pens lined with clean, dry wood shavings to ensure comfort, uniform management, and biosecurity. The experimental treatments consisted of: (1) negative control (no extract supplementation); (2) M100, receiving 200 mg/L *M. oleifera* methanolic extract; (3) T100, receiving 300 mg/L *T. vulgaris* methanolic extract; (4) M50T50, receiving a combination of 100 mg/L *M. oleifera* and 150 mg/L *T. vulgaris* extracts; (5) M75T25, supplemented with 150 mg/L *M. oleifera* and 75 mg/L *T. vulgaris* extracts; and (6) M25T75, receiving 50 mg/L *M. oleifera* and 225 mg/L *T. vulgaris* extracts.

The methanolic extracts were administered via drinking water, which was supplied **ad libitum** throughout the experimental period. To ensure uniform exposure, **all pens were equipped with identical drinkers**, and the extracts were **freshly prepared each day, thoroughly homogenized, and offered at the same concentration to all birds within each treatment group. Water intake was monitored at the pen level on a daily basis by measuring the difference between the volume of water offered and the residual volume**, allowing estimation of average water consumption per bird.

Although individual bird water intake could not be quantified, daily pen-level monitoring indicated no marked differences in water consumption among treatment groups, suggesting comparable extract exposure. Nevertheless, the administration of bioactive compounds via drinking water may introduce some variability in individual intake due to differences in drinking behavior, which represents a potential limitation of the study. This variability may have contributed to minor inter-bird differences in extract consumption, but the consistent and significant treatment effects observed across multiple performance, immunity, and gut health parameters indicate that the overall conclusions regarding the synergistic efficacy of combined *M. oleifera* and *T. vulgaris* extracts remain robust.

A standard corn–soybean meal-based basal diet was formulated to meet or exceed the nutrient requirements for broilers as recommended by National Research Council (1994) and provided in mash form Table 2. The experiment lasted for 42 days, including a 7-day pre-treatment adaptation period during which birds were acclimatized to experimental conditions and management practices.

**Table 2:** Composition of basal feed of experimental birds.

Ingredients (%)	Starter phase (1–21 day)	Finisher phase (22–42 day)
Corn	55.2	59.8
Soybean meal	36.8	24.6
Corn gluten meal	2.10	6.85
Corn oil	2.30	2.65
Limestone	0.78	0.70
Dicalcium phosphate	2.25	2.05
Salt	0.52	0.54
Vitamin-mineral premix a	0.50	0.50
Lysine HCL	0.23	0.38
DL-Methionine	0.20	0.13
Threonine	0.11	0.09
Choline chloride	0.06	0.06
Chemical composition		
Nutrient	Starter phase (1–21 day)	Finisher phase (22–42 day)
Crude protein (%)	23.2	20.9
ME (Kcal/kg)	3010	3160
Methionine (%)	0.53	0.45
Sulphur amino acid (%)	0.91	0.77
Lysine (%)	1.38	1.21
Calcium (%)	1.05	0.89
Phosphorus (%)	0.49	0.47
Threonine (%)	0.94	0.87

Vitamin-mineral premix contains in the following per kg: vitamin A, 2,400,000 IU; vitamin D3, 1,000,000 IU; vitamin E, 16,000 IU; vitamin K, 800 mg; vitamin B1, 600 mg; vitamin B2, 1600 mg; vitamin B6, 1000 mg; vitamin B12, 6 mg; niacin, 8000 mg; folic acid, 400 mg; pantothenic acid, 3000 mg; biotin, 40 mg; antioxidant, 3000 mg; cobalt, 80 mg; copper, 2000 mg; iodine, 400 mg; iron, 1200 mg; manganese, 18,000 mg; selenium, 60 mg; zinc, 14,000 mg.

Throughout the experimental period, all groups were maintained under identical environmental, managerial, and hygienic conditions. The brooding temperature was initially maintained at 33–34°C during the first week and gradually reduced by 2–3°C per week until reaching 24°C by the end of the fourth week. A continuous lighting program (23 h light:1 h dark) was implemented to promote feed intake and growth. Feed and water were provided ad libitum, and all pens were regularly cleaned and disinfected to prevent disease outbreaks.

All management practices, including vaccination schedules and health monitoring, were conducted according to standard commercial guidelines. Any mortality was recorded daily, and general bird behavior and health status were observed throughout the trial to ensure welfare and reliability of experimental data.

### Growth performance and carcass evaluation

Feed intake was recorded daily throughout the experimental period. The feed consumed by each replicate was determined by subtracting the weight of the feed refused from the total feed offered. In other words, feed intake (in grams) was calculated as the difference between the amount of feed offered and the amount of feed left uneaten. This procedure allowed for accurate monitoring of feed consumption trends and ensured consistency across treatment groups. Body weight gain was measured weekly for each replicate. It was calculated by subtracting the initial body weight at the beginning of the week from the final body weight at the end of the same week. Thus, the weekly body weight gain represented the change in live weight during each seven-day period, providing an indicator of growth rate and allowing for comparisons among treatments over time. Feed conversion ratio was used to assess feed efficiency and was expressed as the ratio of total feed intake to total body weight gain for each replicate. In practical terms, FCR was obtained by dividing the total amount of feed consumed by the total body weight gained over the experimental period. A lower FCR value indicated more efficient feed utilization by the birds.

At the end of the feeding trial, dressing percentage was calculated to evaluate carcass yield. Five birds from each replicate were randomly selected and individually weighed to obtain live body weight before slaughter. After slaughtering, feathers, viscera, and other inedible parts were removed, and the carcass (including breast, thigh, leg, and wings) was weighed. Dressing percentage was determined by dividing carcass weight by live body weight and multiplying the result by 100. This value represented the proportion of the edible carcass relative to the live body weight, serving as an indicator of overall production efficiency and meat yield potential in broilers.

### Gut pH

The pH of various sections of the gastrointestinal tract was measured using a digital pen-type pH meter. During slaughter, the electrode was gently inserted into the crop, gizzard, and small intestine containing chyme or digesta, and the pH readings were recorded with care to avoid damaging the tissues (Mabelebele et al., 2017).

### Antibody titer

To evaluate the immune status of the birds, antibody titers were measured against infectious bursal disease (IBD) virus, Newcastle disease (ND) virus, and infectious bronchitis (IB) virus. Approximately 3 mL of blood was collected from the brachial vein using a sterile syringe on days 21 and 42 of the experiment. The collected blood samples were centrifuged to separate the serum, which was then used to determine antibody titers against each virus using a commercial ELISA kit.

### Weight of lymphoid organs

On day 42, at the end of the experimental period, **two birds per pen (replicate)** were randomly selected, individually weighed, and humanely slaughtered. The major lymphoid organs, including the spleen, bursa of Fabricius, and thymus, were carefully excised and weighed **separately for each bird** using a digital balance. **Relative organ weights were calculated for each bird as a percentage of its individual live body weight**, and the **mean of the two birds within each pen was used as the experimental unit for statistical analysis** to assess lymphoid organ development and immune status.

### Liver and kidney function tests

Liver and kidney function tests were performed to assess any potential toxic or adverse effects of the methanolic extracts of *M. oleifera* and *T. vulgaris.* On day 42 of the experiment, approximately 4–5 mL of blood was collected from the wing vein of five birds per treatment group into **EDTA-coated tubes**. The samples were centrifuged at **4000** **rpm for 3 minutes**, and the **plasma** was carefully separated and stored at −20°C until biochemical and antioxidant analyses. Liver function biomarkers, including **alkaline phosphatase (ALP), aspartate aminotransferase (AST), and alanine aminotransferase (ALT)**, were quantified in the **plasma** using commercial diagnostic kits (Manufacturer: **Randox Laboratories, UK**; Catalog no.: **[ALP: NX2336, AST: NX2332, ALT: NX2331]**) and a spectrophotometer (Model: **Thermo Scientific Evolution 201**). Similarly, kidney function parameters, including **urea and creatinine**, were determined using commercial kits (Manufacturer: **Randox Laboratories, UK**; Catalog no.: **Urea: NX2334, Creatinine: NX2333**) following the manufacturer’s instructions. All assays were performed in triplicate to ensure accuracy and reproducibility.

### Nutrients digestibility

For nutrient digestibility, **five birds per replicate** were randomly selected and euthanized via intravenous injection of **sodium pentobarbitone**. To assess apparent nutrient digestibility, **digesta were carefully collected from the distal ileum**, defined as the section of the small intestine between Meckel’s diverticulum and approximately 2 cm proximal to the ileocecal junction. The intestinal segment was gently excised, and its contents were **squeezed into clean, labeled containers** to minimize contamination with mucosa or other intestinal contents. The collected digesta were immediately stored in a **deep freezer (Haier HDF-545DD, China) at 0**°**C** until analysis. Apparent digestibility coefficients of nutrients were estimated using the **chromium oxide (Cr₂O₃) indigestible marker** method. Chromic oxide concentrations were determined using UV-spectrophotometry following wet digestion with perchloric and nitric acids. Following freeze-drying, the dry matter, crude protein, ether extract, crude fibre, and apparent metabolizable energy levels of all the feed and ileal digesta samples were measured using the methodology provided by (Hafeez et al. 2024).

$$\text{Apparent}\;\text{digestibility}=100-\left[100\;\times \frac{\text{Nutreint}\;\left(\%\right)\;\text{in}\;\text{digesta}\times \text{Cr}\left(\%\right)\text{in}\;\text{feed}}{\text{Nutrient}\left(\%\right)\text{in}\;\text{feed}\times \text{Cr}\left(\%\right)\text{in}\;\text{digesta}}\right]$$

Where Cr = chromic oxide

### Statistical analysis

All experimental data were analyzed using **one-way analysis of variance (ANOVA)** to evaluate the effects of different combinations of *M. oleifera* and *T. vulgaris* methanolic extracts on broiler performance, immunity, gut health, and nutrient digestibility. The **experimental unit was the pen for performance data** (feed intake, body weight gain, FCR), whereas **individual birds were considered as experimental units for carcass traits, organ weights, biochemical, and digestibility parameters**. The **main effect** was the treatment (six extract combinations), and **replicates within treatments were treated as random effects**. Prior to analysis, all datasets were examined for **outliers using the Grubbs’ test**, and **normality of residuals was checked using the Shapiro–Wilk test**. When significant differences among treatments were detected (*p* < 0.05), **mean comparisons were performed using Tukey’s Honestly Significant Difference (HSD) post hoc test**. Results are reported as **mean ± standard error of the mean (SEM)**.

## Results

### Growth performance

The effect of *M. oleifera* and *T. vulgaris* methanolic extracts, alone or in combination, on broiler growth performance is presented in Table 3. Feed intake and body weight gain differed significantly among treatments (*p* = 0.01). Birds receiving the combined extract (M50T50) consumed more feed and gained greater weight compared to all other groups, whereas the control birds showed the lowest values. The feed conversion ratio (FCR) was also significantly affected (*p* = 0.01), with birds in the M50T50 and M75T25 groups exhibiting more efficient feed utilization (lower FCR) compared to the control and single-extract treatments. Livability, however, did not vary significantly across treatments (*p* = 0.07), though slightly lower survival was noted in groups receiving single extracts.

**Table 3:** Effect of methonolic extract of moringa olifera and thymus vulgaris alone or in different combinations on body performance on day 42 (*n* = 540).

Groups	Feed intake (g)	Weight gain (g)	Feed conversion ratio	Livability (%)
**Control**	3425.0^d^	1618.9^e^	2.0679^a^	100
M100	3754.7^b^	2032.9^c^	1.7957^b^	73.99
T100	3618.3^c^	1957.5^d^	1.8053^b^	88.88
M50T50	3829.0^a^	2329.2^a^	1.6202^c^	88.88
M75T25	3795.5^ab^	2214.5^b^	1.6794^c^	75.55
M25T75	3650.0^c^	1951.2^d^	1.8194^b^	82.22
SEM	37.56	97.86	0.78	28.65
P. value	0.01	0.01	0.01	0.07

Means in the same row with various superscripts for each treatment are significantly different (*P* < 0.05).

M100: 200 mg/L Moringa.

T100: 300 mg/L Thyme.

M50T50: 100 mg/L Moringa + 150 mg/L Thyme.

M75T25: 150 mg/L Moringa + 75 mg/L Thyme.

M25T75: 50 mg/L Moringa + 225 mg/L Thyme.

### Carcass traits

The effects of *M. oleifera* and *T. vulgaris* extracts on carcass characteristics are shown in Table 4. Birds in the M50T50 group exhibited a significantly higher dressing percentage compared with the control and single-extract groups (*p* = 0.01). Similarly, thigh and breast muscle yields were markedly greater in birds receiving the combined treatments, particularly M50T50 and M75T25, compared to those given M100, T100, or the control (*p* = 0.01). Wing percentage was not significantly influenced by dietary treatments (*p* = 0.05).

**Table 4:** Effect of methonolic extract of moringa olifera and thymus vulgaris alone or in different combinations on dressing percentage and relative weights (%) of selected organs (*n* = 450).

Group	Dressing %	Thigh %	Breast %	Wings %
Control	66.56^d^	17.43^c^	31.24^c^	8.86
M100	69.15^bc^	18.22^b^	32.17^b^	8.58
T100	67.65^cd^	17.89^bc^	32.08^bc^	8.31
M50T50	71.95^a^	20.16^a^	34.36^a^	8.68
M75T25	70.22^ab^	18.50^b^	32.50^b^	9.09
M25T75	68.89^bc^	18.49^b^	32.75^b^	8.98
SEM	4.56	2.98	1.92	1.08
P value	0.01	0.01	0.01	0.05

Means in the same row with various superscripts for each treatment are significantly different (*P* < 0.05).

M100: 200 mg/L Moringa.

T100: 300 mg/L Thyme.

M50T50: 100 mg/L Moringa + 150 mg/L Thyme.

M75T25: 150 mg/L Moringa + 75 mg/L Thyme.

M25T75: 50 mg/L Moringa + 225 mg/L Thyme.

### Gut pH

As presented in Table 5, the dietary inclusion of *M. oleifera* and *T. vulgaris* extracts had no significant impact on crop (*p* = 0.32) and gizzard (*p* = 0.05) pH values. However, significant reductions in intestinal pH were observed in the duodenum (*p* = 0.03), jejunum (*p* = 0.01), and ileum (*p* = 0.01) of birds supplemented with either single or combined extracts compared with the control group. The greatest pH reduction was observed in the M100 and T100 groups, suggesting enhanced intestinal acidification and potentially improved microbial balance.

**Table 5:** Effect of methonolic extract of moringa olifera and thymus vulgaris alone or in different combinations on crop, gizzard and small intestine pH. (*n* = 60).

Group	Crop	Gizzard	Duodenum	Jejunum	Ileum
Control	5.37	6.82	7.02^a^	7.27^a^	7.19^a^
M100	5.63	6.59	6.13^b^	6.18^c^	6.03^c^
T100	5.58	6.61	6.23^b^	6.21^c^	6.03^c^
M50T50	5.27	6.74	6.54^ab^	6.98^ab^	6.18^bc^
M75T25	5.48	6.34	6.42^b^	6.64^bc^	6.19^b^
M25T75	5.37	6.42	6.38^b^	6.72^bc^	6.15^bc^
SEM	0.65	0.32	0.21	0.97	1.03
P value	0.32	0.06	0.03	0.01	0.01

Means in the same row with various superscripts for each treatment are significantly different (*P* < 0.05).

M100: 200 mg/L Moringa.

T100: 300 mg/L Thyme.

M50T50: 100 mg/L Moringa + 150 mg/L Thyme.

M75T25: 150 mg/L Moringa + 75 mg/L Thyme.

M25T75: 50 mg/L Moringa + 225 mg/L Thyme.

### Immune response

The antibody titers against infectious bronchitis (IB), Newcastle disease (ND), and infectious bursal disease (IBD) are shown in Table 6. Significant improvements were observed in antibody titers across all viruses at both 21 and 42 days (*p* < 0.01). Birds supplemented with the combined extracts, especially M50T50, exhibited markedly higher titers compared to both the control and single-extract groups. Among the individual extracts, *M. oleifera* (M100) produced a stronger antibody response than T*. vulgaris* (T100), while the control birds consistently recorded the lowest immune response throughout the trial.

**Table 6:** Effect of methonolic extract of moringa oleifera and thymus vulgaris alone or in different combinations on immunity (*n* = 60).

Groups	Antibody titer against IB		Antibody titer against ND		Antibody titer against IBD	
	Day-21	Day-42	Day-21	Day-42	Day-21	Day-42
Control	4.66^c^	4.52^c^	4.52^c^	4.47^c^	1594.0^c^	1218.9^c^
M 100	5.70^b^	5.64^b^	5.58^b^	5.56^b^	1638.2^b^	1227.5^b^
T 100	5.58^b^	5.62^b^	5.50^b^	5.54^b^	1617.4^bc^	1222.3^bc^
M50 T50	6.47^a^	6.56^a^	6.43^a^	6.48^a^	1687.4^a^	1236.3^a^
M75T25	5.65^b^	5.69^b^	5.57^b^	5.61^b^	1657.9^ab^	1230.3^ab^
M25T75	5.80^b^	5.67^b^	5.72^b^	5.52^b^	1655.4^ab^	1228.7^ab^
SEM	0.45	0.67	0.33	0.42	35.44	10.98
p. value	0.01	0.01	0.01	0.01	0.01	0.01

Means in the same row with various superscripts for each treatment are significantly (*P* < 0.05).

M100: 200 mg/L Moringa.

T100: 300 mg/L Thyme.

M50T50: 100 mg/L Moringa + 150 mg/L Thyme.

M75T25: 150 mg/L Moringa + 75 mg/L Thyme.

M25T75: 50 mg/L Moringa + 225 mg/L Thyme.

### Lymphoid organ weight

The relative weights of major lymphoid organs are presented in Table 7. The bursa of Fabricius weight differed significantly among treatments (*p* = 0.01), with birds in the M50T50 group showing the highest relative bursal weight compared to the control and other groups. However, spleen (*p* = 0.51) and thymus (*p* = 0.05) weights did not vary significantly, though numerically higher values were noted in the combined-extract groups, indicating a trend toward improved immune organ development.

**Table 7:** Effect of methonolic extract of moringa oleifera and thymus vulgaris alone or in different combinations on lymphoid organs weight (*n* = 60).

Groups	Spleen%	Bursa%	Thymus%
Control	0.08	0.10^c^	0.26
M100	0.11	0.12^ab^	0.32
T100	0.09	0.13^bc^	0.28
M50T50	0.10	0.18^a^	0.36
M75T25	0.11	0.16^ab^	0.34
M25T75	0.12	0.14^bc^	0.35
SEM	0.03	0.05	0.09
P. value	0.51	0.01	0.05

Means in the same row with various superscripts for each treatment are significantly different at α=0.05.

M100: 200 mg/L Moringa.

T100: 300 mg/L Thyme.

M50T50: 100 mg/L Moringa + 150 mg/L Thyme.

M75T25: 150 mg/L Moringa + 75 mg/L Thyme.

M25T75: 50 mg/L Moringa + 225 mg/L Thyme.

### Liver and kidney function

As shown in Table 8, supplementation with *M. oleifera* and *T. vulgaris* extracts had no significant effect on liver enzymes (ALT, AST, and ALP) or kidney function markers (uric acid and creatinine). Birds receiving the combined extracts, particularly M50T50 and M75T25, exhibited slightly lower ALT and AST activities than the control, suggesting a hepatoprotective effect without any evidence of toxicity.

**Table 8:** Effect of methonolic extract of moringa oleifera and thymus vulgaris alone or in different combinations on liver and kidney function (*n* = 60).

GROUP	ALT (IU/L)	AST(IU/L)	ALP (IU/L)	Uric acid (mg/dL)	Critinine (mg/dL)
Control	22.04	54.21	86.18	3.86	0.77
M 100	14.27	50.403	83.04	2.85	0.68
T 100	17.90	46.140	85.76	2.01	0.79
M50 T50	15.08	47.15	80.34	2.37	0.58
M75T25	14.57	39.58	79.85	3.14	0.60
M25T75	14.09	53.19	80.91	3.56	0.71
SEM	5.65	7.34	1.03	1.02	0.23
P value	0.05	0.14	0.31	0.06	0.11

M100: 200 mg/L Moringa.

T100: 300 mg/L Thyme.

M50T50: 100 mg/L Moringa + 150 mg/L Thyme.

M75T25: 150 mg/L Moringa + 75 mg/L Thyme.

M25T75: 50 mg/L Moringa + 225 mg/L Thyme.

### Nutrient digestibility

The results for nutrient digestibility are summarized in Table 9. Among all parameters, only crude protein digestibility showed a significant improvement (*p* = 0.01), with the highest values observed in birds supplemented with M50T50, followed by M75T25, compared to the control and single-extract groups. Digestibility of dry matter, ether extract, crude fiber, ash, and nitrogen-free extract remained statistically unaffected (*p* > 0.05), although slight numerical improvements were evident in the combined-extract treatments.

**Table 9:** Effect of methonolic extract of moringa oleifera and thymus vulgaris alone or in different combinations on Nutrient Digestibility (%) (*n* = 60).

Treatment	Dry matter	Ash		Crude protein		Ether extract	Crude fiber	Nitrogen free extract
Control	69.35	43.56	70.37c		77.04		82.89	80.43
M100	70.28	44.66	72.26ab		78.82		83.56	81.55
T100	69.59	43.89	70.79c		78.60		81.63	80.88
M50T50	71.78	43.72	73.01a		80.08		83.99	82.59
M75T25	70.66	44.99	71.95ab		79.77		83.62	81.75
M25T75	70.10	44.72	71.20bc		77.19		81.29	80.45
SEM	1.67	1.02	2.56		1.65		1.44	0.78
P value	0.14	0.34	0.01		0.12		0.29	0.06

Means in the same row with various superscripts for each treatment are significantly different (*P* < 0.05).

M100: 200 mg/L Moringa.

T100: 300 mg/L Thyme.

M50T50: 100 mg/L Moringa + 150 mg/L Thyme.

M75T25: 150 mg/L Moringa + 75 mg/L Thyme.

M25T75: 50 mg/L Moringa + 225 mg/L Thyme.

## Discussion

The present study evaluated the effects of *M. oleifera* and *T. vulgaris* methanolic extracts, singly and in combination, on broiler growth performance and carcass traits. The results showed that the combined supplementation, particularly at 100 mg/L *M. oleifera* and 150 mg/L *T. vulgaris* (M100T150), produced superior growth and feed efficiency compared with the control and single-extract treatments. This synergistic effect may be attributed to the complementary bioactive profiles of both plants. *M. oleifera* contains flavonoids, phenolic acids, and glucosinolates with potent antioxidant and antimicrobial properties, while *T. vulgaris* provides thymol and carvacrol, which possess strong antimicrobial and metabolic-stimulating effects (Abdelli et al., 2021; Oni et al., 2025).

Comparable to the findings of Cui et al. (2018), who reported that dietary *M. oleifera* leaf inclusion enhanced meat oxidative stability and improved redox status without adverse effects on growth, the present results suggest that moderate inclusion levels of *M. oleifera* extract promote metabolic efficiency. However, unlike the growth-suppressive effects observed at high dietary inclusion levels (≥5%) by Cui et al. (2018), the lower aqueous concentrations used in this study appeared beneficial, highlighting a dose-dependent response. This difference likely arises from the controlled extract concentration, which optimizes antioxidant activity without depressing feed intake or nutrient digestibility.

The improved FCR in the combined groups could also be linked to enhanced gut morphology and microbial balance. Thymol and carvacrol stimulate digestive enzyme secretion and promote the proliferation of *Lactobacillus* spp. while suppressing *E. coli* and other enteropathogens (Banday et al., 2025). This balanced gut microbiota supports villus development and efficient nutrient absorption, thereby improving feed utilization. Additionally, the antioxidant components of both extracts may mitigate lipid peroxidation and enhance nutrient metabolism, ultimately leading to improved carcass yield and meat quality.

Birds receiving the combined extracts, particularly M100T150, exhibited higher dressing percentages and breast muscle yields than controls and individual extract groups. These improvements may be linked to enhanced nutrient absorption, protein retention, and improved gut function due to reduced pathogen load. Higher breast yield is associated with improved amino acid utilization, likely mediated by *M. oleifera* glucosinolates and flavonoids stimulating metabolism, and thymol from thyme enhancing nitrogen retention (Akib et al., 2024; Jiang et al., 2023). The dose effect observed, with M100T150 outperforming higher levels of individual extracts, suggests that intermediate combinations optimize physiological benefits without causing metabolic stress.

The intestinal pH results show significant acidification in duodenum/jejunum/ileum in supplemented birds (particularly single-extract M100 and T100 and combined groups). Phytogenics commonly alter luminal pH and microbiota composition: essential oils such as thymol/carvacrol suppress acid-sensitive pathogens and selectively enrich commensals that lower luminal pH (e.g., lactic acid bacteria), while polyphenols and tannins in Moringa influence microbial fermentation end-products (short-chain fatty acids) that acidify the luminal contents (Oni et al., 2025; Abdelli et al., 2021). Lower small-intestinal pH promotes proteolytic enzyme activity and reduces proliferation of opportunistic Enterobacteriaceae, which in turn improves digestibility and nutrient absorption—mechanisms that match our finding of improved crude-protein digestibility in treated birds.

Only crude protein digestibility was significantly improved, with the M50T50 group showing the highest value. This finding is consistent with Egbu et al. (2022), who reported enhanced protein utilization in broilers receiving *M. oleifera* seed extract via drinking water. Given that the majority of protein digestion and amino acid absorption occur in the **upper small intestine**, the observed improvement is more plausibly attributed to **enhanced pre-cecal digestive efficiency** rather than direct effects of microbial suppression in the lower gut. Bioactive compounds present in *M. oleifera* and *T. vulgaris* may stimulate endogenous digestive processes by modulating intestinal pH and promoting the secretion or activity of proteolytic enzymes, thereby improving protein hydrolysis and amino acid availability before the terminal ileum (Oni et al., 2025).

While suppression of proteolytic pathogens in the distal gut is unlikely to directly influence pre-cecal amino acid absorption, it may indirectly support overall protein utilization by reducing post-ileal nitrogen losses and limiting microbial deamination of undigested protein fractions. In addition, the strong antioxidant properties of moringin, thymol, and carvacrol **may contribute to the preservation of intestinal epithelial integrity**, which could support villus architecture and absorptive capacity in the small intestine; however, **direct measurements of villus height or surface area were not performed in the present study**, and this proposed mechanism remains speculative. The lack of significant effects on ether extract and dry matter digestibility suggests that the combined extracts exerted a **selective influence on protein digestion and assimilation**, rather than broadly altering nutrient utilization, which aligns with earlier observations reported by Egbu et al. (2022).

Antibody titers against infectious bronchitis (IB), Newcastle disease (ND), and infectious bursal disease (IBD) were significantly elevated in all extract-supplemented groups, with the strongest responses observed in the M50T50 group at both 21 and 42 days. This enhanced humoral immunity indicates that the combined supplementation of *M. oleifera* and *T. vulgaris* methanolic extracts effectively potentiated immune competence in broilers. The immunostimulatory effect is attributable to the rich bioactive composition of both plants. *M. oleifera* provides vitamins A, C, and E, as well as flavonoids and polyphenols, while *T. vulgaris* contains thymol, carvacrol, and isocaryophyllene, which collectively enhance lymphocyte proliferation, macrophage activity, and cytokine expression (Akib et al., 2024; Abdel-Ghaney et al., 2017).

The elevated antibody titers observed in this study are consistent with earlier findings. Abdel-Ghaney et al. (2017) reported that dietary inclusion of 5–15 g/kg *T. vulgaris* powder significantly increased serum IgG, IgM, IFN-γ, and IL-10 levels, in addition to improving antioxidant status through elevated glutathione and superoxide dismutase (SOD) activities. Similarly, Ahmadian et al. (2020) demonstrated that thyme supplementation enhanced antibody titers against ND and influenza, indicating strengthened humoral and cellular immune responses. These outcomes support the immunomodulatory potential of thyme-derived phenolic monoterpenes and isocaryophyllene, which stimulate cytokine-mediated lymphocyte activation and reduce lipid peroxidation in immune tissues, thereby improving both the efficiency and resilience of the immune system.

The increased bursal weight and enhanced thymic and splenic indices observed in the combined-treatment groups further validate the serological findings, suggesting stimulation of both primary and secondary lymphoid organ development. This organotropic response may result from enhanced differentiation and proliferation of lymphoid cells driven by phytogenic bioactives that upregulate interleukin signaling and promote leukopoiesis (Abdelli et al., 2021). The role of *M. oleifera* in improving lymphoid tissue architecture is well-documented; its potent antioxidant constituents mitigate oxidative stress in developing lymphocytes and support the maturation of immune organs. Moreover, the synergistic effect of *M. oleifera* flavonoids and thyme phenolics likely amplifies antioxidant defenses in lymphoid tissues, as both extracts inhibit lipid peroxidation and preserve the redox balance essential for lymphocyte viability (Jan et al., 2025).

The observed enhancement in antibody response and lymphoid organ development can be mechanistically attributed to a combination of antioxidant protection, cytokine modulation, and gut–immune axis regulation. The antioxidant constituents in both extracts neutralize free radicals, protecting immune cells from oxidative apoptosis and maintaining the structural integrity of lymphoid tissues. Concurrently, thymol and carvacrol from thyme stimulate macrophage activation and interleukin-2 secretion, which strengthen both antibody synthesis and cell-mediated immunity. Additionally, improved intestinal health due to the antimicrobial and prebiotic actions of phytochemicals enhances nutrient absorption and antigen presentation, which collectively promote a more efficient immune response (Ahmadian et al., 2020; Abdel-Ghaney et al., 2017).

Hepatic and renal markers. Liver enzymes (ALT, AST, ALP) and kidney markers (urea, creatinine) were not significantly altered across treatments, indicating no evidence of hepatotoxicity or renal impairment at the tested doses. This finding is important because some concentrated essential-oil preparations can be hepatotoxic at high doses; however, the methanolic extracts and the doses used here appear safe and may even show hepatoprotective trends (numerically lower ALT/AST) in combined groups—an effect previously attributed to antioxidant constituents in Moringa (Akib et al., 2024). Nevertheless, widely-documented dose-dependent toxicity for concentrated thyme essential oil cautions that therapeutic windows must be respected in further work.

The superior response observed with the mid-level combination of *M. oleifera* and *T. vulgaris* extracts may be explained by a **complementary rather than singular mode of action**, although the exact mechanisms were not directly measured in the present study. Thyme-derived phenolic compounds, such as thymol and carvacrol, are widely reported to exert antimicrobial effects against enteric pathogens, while *M. oleifera* is recognized for its high content of antioxidant polyphenols and micronutrients that support physiological and immune functions in poultry (Abdelli et al., 2021). The combined use of both extracts at moderate inclusion levels may therefore allow biological activity to be maintained **without excessive exposure to any single bioactive compound**, potentially reducing the risk of adverse effects associated with higher dosages of individual phytogenics. In addition, phytogenic combinations have been shown to exert broader biological effects than single additives by influencing multiple digestive and metabolic pathways simultaneously, which may partially explain the improved growth performance and protein utilization observed in the combined-extract groups (Oni et al., 2025). However, further studies incorporating targeted microbial, enzymatic, and molecular analyses are required to confirm these interactions.

While the data show clear benefits, mechanistic confirmation would be strengthened by direct measures of gut microbiota composition, digestive enzyme activities (e.g., pancreatic protease, brush-border peptidases) and oxidative stress biomarkers in tissues. Dose-response trials and fractionation (isolating thymol, carvacrol, key flavonoids) would help determine the active fractions and safety margins. Finally, longer-term trials and challenge models with defined pathogens would clarify the robustness of the immunoprotective effects observed here (Ali et al., 2025; Oni et al., 2025).

## Conclusion

The study demonstrates that dietary supplementation of *M. oleifera* and *T. vulgaris* methanolic extracts, particularly in combination at M50T50, significantly enhances growth performance, feed efficiency, carcass yield, immune response, and crude protein digestibility in broilers. Combined supplementation also positively influenced intestinal pH and showed a trend toward improved lymphoid organ development without causing any adverse effects on liver or kidney function. These findings indicate that the synergistic use of *M. oleifera* and *T. vulgaris* extracts can serve as an effective and safe strategy to improve broiler productivity and health.

## Funding

We extend our appreciation to the Ongoing Research Funding (ORF-2026-833), King Saud University, Riyadh, Saudi Arabia.

## Institutional review board statement

All the materials and methods were pre-approved from the Faculty of Animal Husbandry and Veterinary Science Ethical Committee through notification No. 6780-A/LM, 10 February 2023.

## Data availability statement

The relevant data are provided in the paper. The data of the current experiment can be obtained from corresponding author when needed.

## Use of artificial intelligence

AI (chatgpt) has been used for English language.

## CRediT authorship contribution statement

**Majid Ali:** Conceptualization, Writing – review & editing, Project administration. **Hanan Alkhalaifah:** Funding acquisition. **Naila Chand:** Conceptualization, Methodology, Writing – original draft, Supervision, Project administration. **Shabana Naz:** Methodology, Writing – original draft. **Rifat Ullah Khan:** Investigation, Data curation, Formal analysis, Writing – original draft. **Ala Abudabos:** Writing – review & editing, Funding acquisition. **Kasim Sakran Abass:** Investigation, Funding acquisition. **Sohail Ahmad:** Investigation, Data curation, Formal analysis. **Ibrahim A. Alhidary:** Writing – review & editing, Funding acquisition.

## Disclosures

The authors declare no competing interest.
